# Toward a Functional Trait Approach to Bee Ecology

**DOI:** 10.1002/ece3.70465

**Published:** 2024-10-18

**Authors:** Madeleine M. Ostwald, Victor H. Gonzalez, Carrie Chang, Nydia Vitale, Mariano Lucia, Katja C. Seltmann

**Affiliations:** ^1^ Cheadle Center for Biodiversity & Ecological Restoration University of California Santa Barbara California USA; ^2^ Undergraduate Biology Program and Department of Ecology and Evolutionary Biology University of Kansas Lawrence Kansas USA; ^3^ Instituto Argentino de Investigaciones de Las Zonas Áridas, CONICET Mendoza Argentina; ^4^ División Entomología, Laboratorio Anexo Museo de La Plata Universidad Nacional de La Plata, CONICET La Plata Argentina

**Keywords:** open science, phenotype, pollinators, trait data

## Abstract

Functional traits offer an informative framework for understanding ecosystem functioning and responses to global change. Trait data are abundant in the literature, yet many communities of practice lack data standards for trait measurement and data sharing, hindering data reuse that could reveal large‐scale patterns in functional and evolutionary ecology. Here, we present a roadmap toward community data standards for trait‐based research on bees, including a protocol for effective trait data sharing. We also review the state of bee functional trait research, highlighting common measurement approaches and knowledge gaps. These studies were overwhelmingly situated in agroecosystems and focused predominantly on morphological and behavioral traits, while phenological and physiological traits were infrequently measured. Studies investigating climate change effects were also uncommon. Along with our review, we present an aggregated morphological trait dataset compiled from our focal studies, representing more than 1600 bee species globally and serving as a template for standardized bee trait data presentation. We highlight obstacles to harmonizing this trait data, especially ambiguity in trait classes, methodology, and sampling metadata. Our framework for trait data sharing leverages common data standards to resolve these ambiguities and ensure interoperability between datasets, promoting accessibility and usability of trait data to advance bee ecological research.

## Graphical abstract

Functional traits are increasingly being used as a tractable framework for understanding bee community ecology and responses to environmental change. Here, we outline best practices for trait measurement and standardization in line with open science principles, to facilitate large‐scale trait data sharing to advance bee functional ecology.

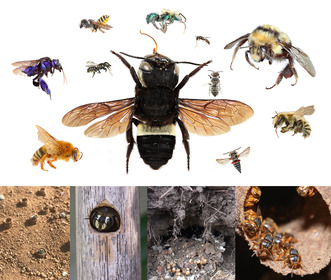

## 1 Introduction

Inferring generalizable patterns in species dynamics, distributions, and functional variation are central aims of ecology and evolutionary biology (MacArthur 1972). Trait‐based approaches, which quantify phenotypic characteristics that impact organisms' fitness and/or functional role, provide a tractable comparative framework for understanding communities, ecosystems, and evolutionary processes (Mcgill et al. 2006; Schleuning, García, and Tobias 2023; Violle et al. 2007). Functional trait studies have proliferated over the past two decades, addressing foundational questions in community ecology (Cadotte et al. 2015; Mcgill et al. 2006; Violle and Jiang 2009), biogeography (Violle et al. 2014), and conservation biology (Cadotte, Carscadden, and Mirotchnick 2011; Wellnitz and Poff 2001) across taxonomic groups. For example, trait studies have clarified ecosystem‐level impacts of biodiversity loss by linking organismal traits to ecosystem functioning (Ali et al. 2023; Carmona et al. 2021). These works emphasize the promise of trait‐based research for generating novel insights into central ecological concepts and theories.

**FIGURE 1:**
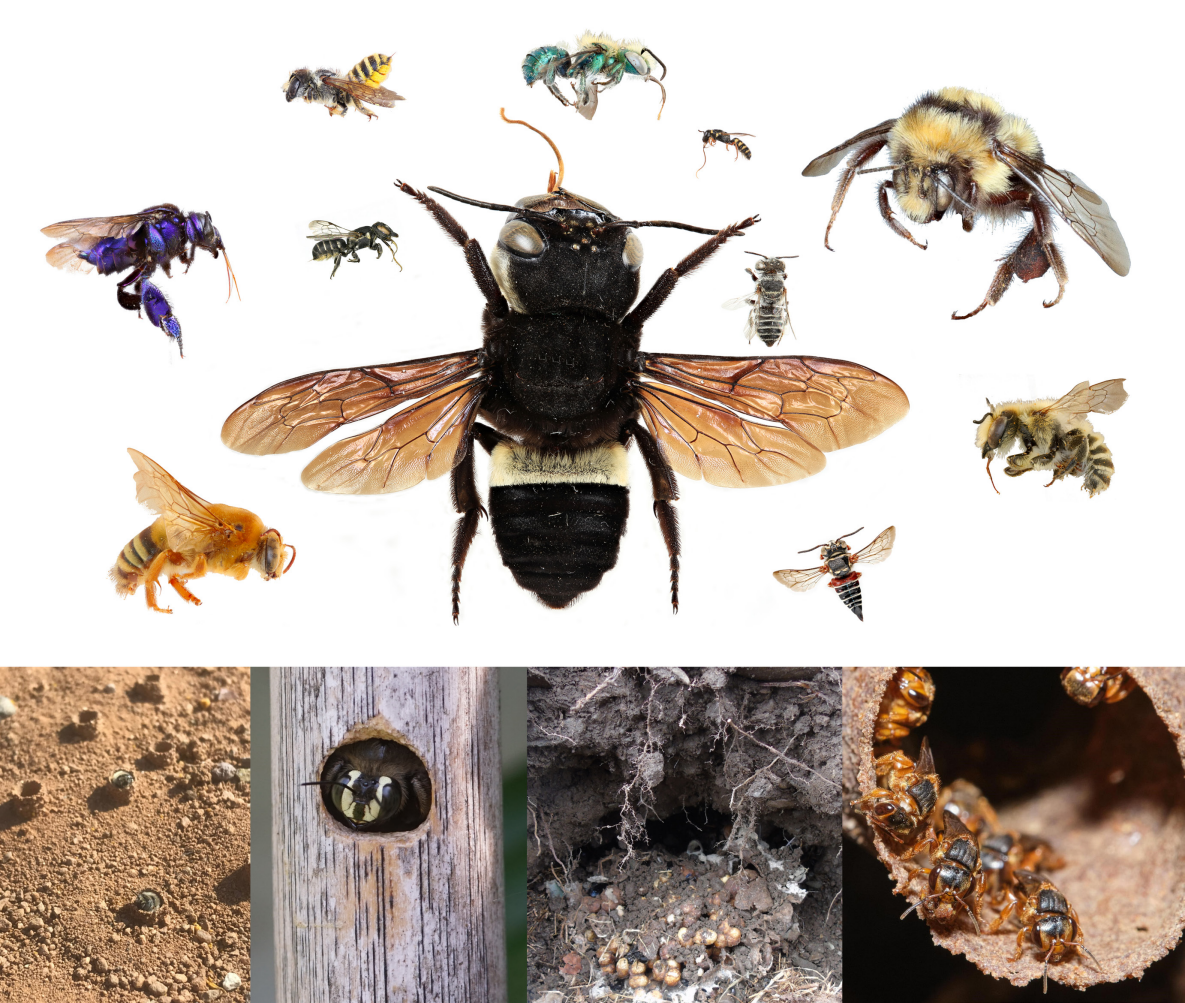
Bees represent an impressive diversity of functional trait states, varying dramatically in morphology (e.g., size, coloration, pilosity, tongue length); (top) and behavior (e.g., nesting biology); (bottom).

However, the scale of trait‐based research is strongly limited by our ability to synthesize trait observations across disparate and heterogeneous data sources. Increasingly, ecologists have called for the development of ecological trait data standards and the application of open science principles to functional trait research (Gallagher et al. 2020; Keller et al. 2023; Schneider et al. 2019). Nevertheless, adoption of these practices has not kept pace with the massive proliferation of trait datasets across study systems. In particular, trait research on bees could benefit from the implementation of a robust framework for standardized data collection and sharing. Bees (Hymenoptera: Apoidea: Anthophila) represent more than 20,000 species worldwide and display dramatic interspecific variation in morphology (Figure 1), behavior, physiology, and phenology, including traits that mediate pollination services and responses to global environmental change (Table S1). Exploration of functional traits has long been a cornerstone of bee research, yet only recently have these traits been systematically applied in bee ecological studies as a comparative framework for understanding community‐level processes. Given their major functional role as the primary animal pollinators of terrestrial ecosystems (Ollerton, Winfree, and Tarrant 2011), bees represent a group ripe for exploration through a functional ecological lens (Greenop, Woodcock, and Pywell 2023).

Here, we review an emerging body of literature that assesses functional traits across bee communities to address questions in bee ecology. We review the variety of methods used to quantify bee trait variation, highlight common methodological problems and inconsistencies, and recommend best practices. We describe geographic, taxonomic, and trait biases across the body of bee functional trait work and highlight research areas that merit particular attention in the future studies. Finally, we emphasize the value of open trait data sharing, and propose community data standards for bee functional trait research to facilitate data reuse. As an initial effort toward large‐scale trait data sharing, we present a harmonized dataset from the studies in our review, comprising nearly 12,000 morphological measurements from over 1600 bee species.

## 2 Trends, Methods, and Biases in Bee Functional Trait Research

To survey the literature on bee functional traits, we searched for publications on the ISI Web of Science, the SciELO database, and Google Scholar using the search strings “bee traits” and “bee functional traits.” We screened the resulting publications to include only those that conformed to our definition of a functional trait approach to an ecological question, that is, which analyzed multiple functional traits comparatively across a sample of multiple bee species in a given environmental context. Correspondingly, we excluded the large body of studies that report trait data for single bee species or single traits, which do not use functional traits as a comparative framework. Such studies include taxonomic revisions and descriptive natural history studies and likely number in the thousands, and while they are beyond the scope of the present review, we emphasize that these studies should be leveraged to populate trait datasets on a question‐driven basis (e.g., how does climate predict body size variation?). We additionally searched through the cited literature in these publications for additional papers that fit our inclusion criteria. We extracted metadata from each of these publications, including information on the focal traits measured, the authors' definitions of these traits, and the sources of trait data (Table S2). All reviewed publications are listed in the Data S1.

**FIGURE 2:**
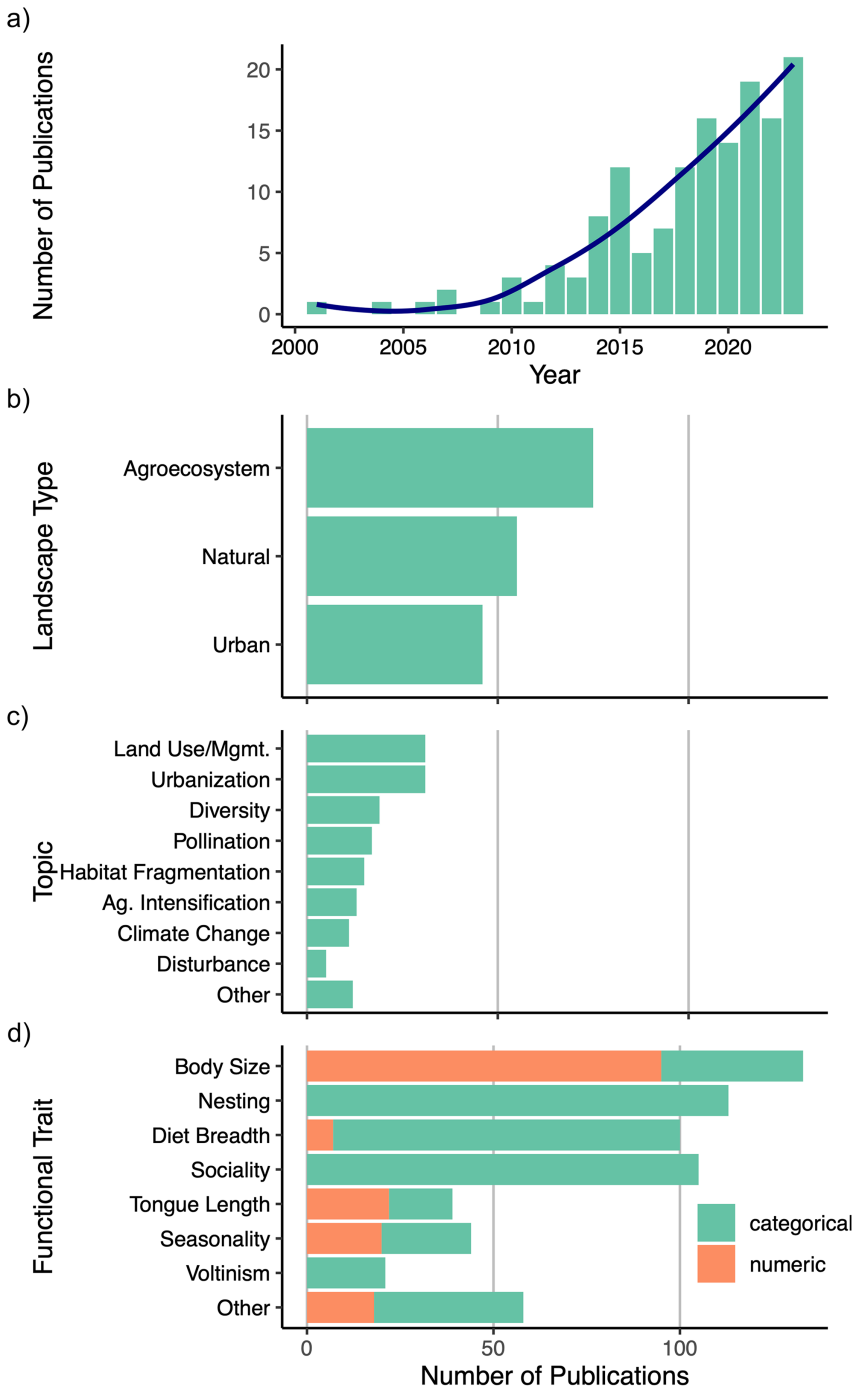
Descriptive metadata from 152 functional trait studies in bee ecology, including (a) the number of bee functional trait studies published each year (excluding publications in the analysis from early 2024), the distribution of publications across (b) landscape contexts and (c) research topics, and (d) the frequencies of focal functional traits in these studies. Studies may assess multiple traits, topics, or landscapes, so the total number of publications sums to greater than the 152 publications analyzed.

**FIGURE 3:**
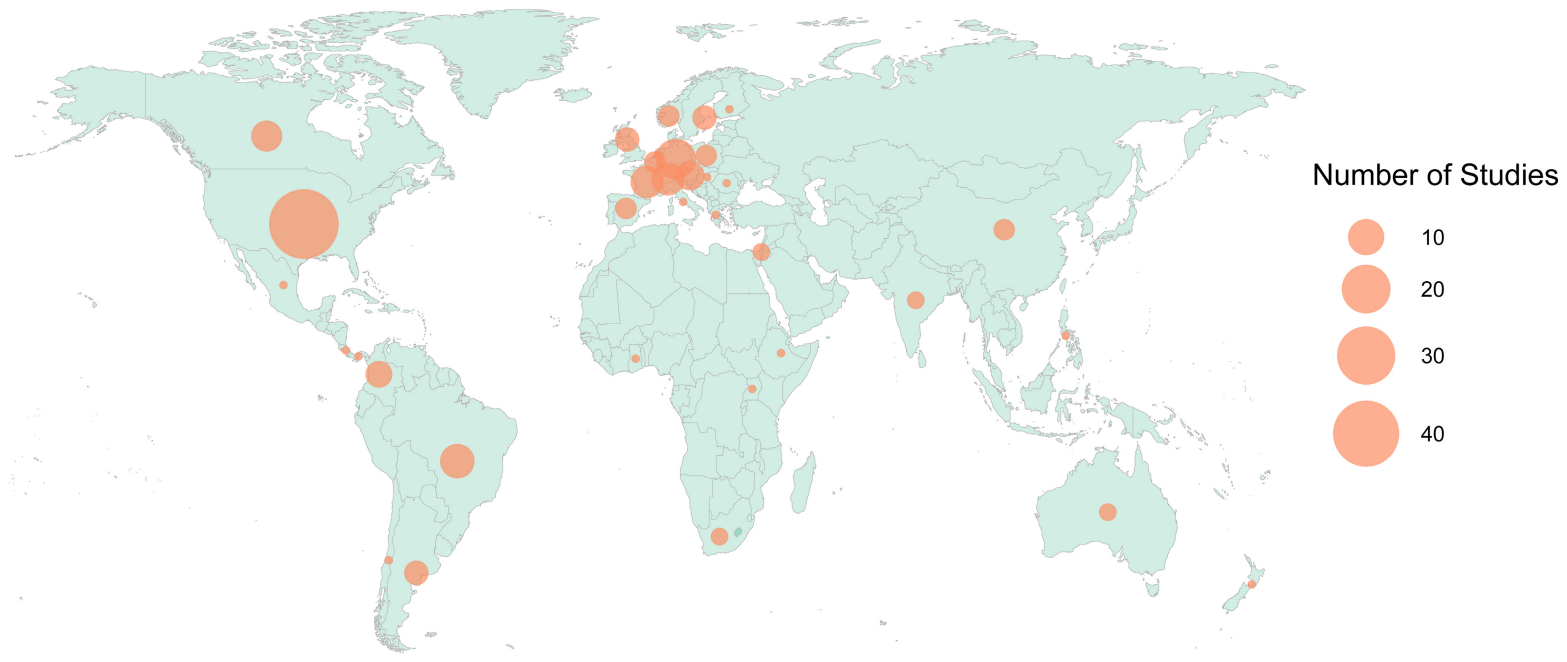
Geographic biases in bee functional trait studies. Circle size indicates the number of published studies assessing traits of bees sampled from a given country. Large‐scale meta‐analyses of existing published functional trait studies across countries are excluded from this map, to prevent double‐counting.

We found and analyzed 152 papers assessing bee functional traits comparatively across species, published between 2001 and May 2024 (Figure 2a; Table S2). Half of the studies examined bees in agroecosystems (58 studies; 49.3%); the remainder were divided between natural landscapes (55 studies; 36.2%), urban landscapes (46 studies; 30.3%), with several studies comparing multiple landscape types (thus the total sums to > 100%); (Figure 2b). The focal topics of these studies were highly variable, but with a particular emphasis on landscape change (e.g., urbanization, habitat fragmentation, land management) (Figure 2c). These studies sampled bees from 35 different countries but were overwhelmingly conducted in the Global North, especially the United States and Northern Europe (116 studies; 76.3%); (Figure 3).

On average, each study quantified 4.29 functional traits (range = 2–10; SE = 0.14) across a sample of 125.7 bee species (range = 2–1460; std. error = 13.26). The most commonly studied functional traits were body size (in 133 or 87.5% of studies), nesting biology (either nesting location, nesting ability, or both; in 113 or 74.3% of studies), diet breadth (100 studies; 65.8%), and sociality (105 studies; 69.1%). Also common were measurements of tongue length (39 studies; 25.7%), seasonality (44 studies; 28.9%), and voltinism (21 studies; 13.8%). Over a third of the studies (58 studies; 38.2%) assessed other, less common functional traits, including measures of pilosity (hairiness), foraging range (often estimated from body size), colony size, native vs. exotic status, reproductive strategy (parasitism), and the use of different pollen‐carrying structures (Figure 2d). Studies sourced their trait data from the literature and published data records (131 studies; 86.2%), from their own measurements and observations (77 studies; 50.7%), and, less commonly, as estimations from allometric equations (19 studies; 12.5%). Below, we discuss measurement strategies, terminology, and possible quantification pitfalls for the most commonly assessed traits in these studies (best practices summarized in Table 1).

**TABLE 1:** Summarized best practices in the collection and sharing of bee functional trait data.

Recommended practice	References
**Trait data collection**	
*Morphological traits*: Where possible, adhere to prevailing measurement protocols to conform with existing datasets (e.g., ITD for body size). When empirical measurements are not possible, estimate via robust allometric equations (e.g., for tongue size). Avoid common measurement pitfalls (e.g., body length) and reporting data only as categorical assignments (e.g., “short” vs. “long”). Prioritize data collection for lesser studied traits, including pilosity and coloration	Cane (1987), Cariveau et al. (2016), Kendall et al. (2019), Roquer‐Beni et al. (2020), Stavert et al. (2016)
*Behavioral traits* (sociality, nesting, diet breadth, etc.): Rigorously define trait classes. Consider adopting existing term structures (see refs.), and cite these	Cane and Sipes (2006), Michener (1974)
*Physiological traits*: Prioritize data collection for these lesser studied traits, including thermal tolerance and desiccation resistance. Consider conforming methodologies (e.g., temperature ramping rates) with existing studies to support meta‐analysis, where appropriate	Roeder, Roeder, and Bujan (2021)
*Phenological traits*: Prioritize data collection for these lesser studied traits, including timing of reproduction, voltinism, flight activity, and overwintering life stage	
**Trait data sharing**	
*Make trait data accessible* at the time of publication. Archive datasets (e.g., through Zenodo, Data Dryad, etc.). Share data in nonproprietary, machine‐readable file formats (e.g., .csv files rather than in .docx or .pdf files)	Wilkinson et al. (2016)
*Share raw data*, not just summarized data. For example, report ITD measurements for all specimens, not just mean values by species. Link raw data to specimen data (e.g., sex, life stage, and sampling location) and accession numbers	
*Include clear metadata* (e.g., measurement protocols, sampling coordinates) alongside raw trait data	
*Report data provenance* to properly credit researchers and to prevent pseudoreplication in future meta‐analyses. Cite sources for all data collected in prior studies, including categorical traits (e.g., "nesting data compiled from Michener et al., 2007")	
*Map trait data to existing data standards* (e.g., Darwin Core for sampling data, GBIF for taxonomic data, HAO for bee morphological data)	Yoder et al. (2010)
*Register trait data* (e.g., through the Open Traits Network)	Gallagher et al. (2020)

### 2.1 Body Size

Body size is among the most widely studied functional traits across animal taxa. Because it correlates with important life history, physiological, and behavioral attributes (e.g., growth rates, lifespan, and fecundity); (Angilletta, Steury, and Sears 2004; Blueweiss et al. 1978; Glazier 2008; Woodward et al. 2005), body size is often a strong predictor of macroecological patterns (Blackburn and Gaston 1994; Chown and Gaston 2010; Peters 1983). Further, in bees, links between body size and pollination traits suggest important functional consequences of size variation at the ecosystem scale (De Luca et al. 2019; Benjamin, Reilly, and Winfree 2014; Jauker, Speckmann, and Wolters 2016). In the studies we surveyed, body size was primarily estimated from the distance between wing pads (tegulae) that cover the base of the forewing (intertegular distance or ITD; in 90 of 133 studies or 67.7%). Intertegular distance is simple to measure and has predominated as a method for estimating bee body size since Cane (1987) established the allometric relationship between this measure and dry mass in female specimens of 20 North American bee species. The ubiquity of ITD measurement in bee ecology could enable meta‐analyses of size effects in different ecological contexts, where raw data are available. However, caution should be taken to address confounding effects of sexual dimorphism and other sources of intraspecific variation in body size. Recently, Kendall et al. (2019) revisited the question of ITD as a proxy for body size and found that this metric is a robust predictor of interspecific size variation when the effects of phylogeny, sex, and biogeography are accounted for. These predictive allometric models are available in the *R* package “pollimetry” made available by the authors, which was used by several studies in our analysis to improve size estimates from ITD (Chase et al. 2023; Kammerer et al. 2021; Kazenel et al. 2024; Kendall et al. 2022). Efforts like these to account for variation in body size can help improve the predictive power of size proxies, especially considering practical constraints of obtaining direct mass measurements from specimens (e.g., due to damage to older specimens or the error introduced by curatorial practices involving glue or pins). Still, we emphasize the advantages of ITD over other, less accurate size proxies such as body length (measured in 20 of 133 studies quantifying body size; 15.0%), which is affected by telescoping of abdominal segments. About a quarter of studies (38 of 133 studies; 28.5%) analyzed size categorically (e.g., “small,” “medium,” and “large”), with size classes reflecting grouped ITD or body length measurements. Less commonly, size classes were made subjectively in reference to a standard (e.g., relative to the size of a honey bee), a practice we discourage in favor of objective measurements. Where data are presented categorically, they should be accompanied by numeric measurements to facilitate data reuse.

### 2.2 Nesting Biology

Nesting biology is highly variable in bees, with consequences for habitat preferences and exposure to environmental stressors. For example, ground‐nesting bees may be better insulated from extreme temperatures and fire than are twig‐ or wood‐nesting bees, yet may be more vulnerable to habitat loss under certain environmental pressures (e.g., urbanization). As such, nesting traits have figured prominently in bee functional trait studies (Figure 2d). Most studies reviewed here categorized nesting biology at the species level according to nest location (e.g., “ground,” “cavity,” and “stem” ), though others additionally or alternatively considered nesting ability, that is, whether a given species excavates its own nest or occupies pre‐existing cavities. More so than other categorical traits such as sociality and diet breadth, nesting trait analysis prompted the use of a large number of unique terms, reflecting the sheer diversity of bee nesting strategies, especially when considering data on tropical bees (e.g., nesting in termite mounds or in exposed nests, Cabral Borges et al. 2020; Giannini et al. 2020; Munyuli 2014; Table S3). By far the most common method of assigning bee species to nesting categories was by distinguishing broadly between aboveground and belowground nesters. Importantly, several authors create categories (e.g., “variable” or “mixed”) that account for within‐species variation in nesting location, for example, bumble bees that can nest either in belowground soil cavities or aboveground tree cavities. Nesting information was predominantly sourced from the literature, and only about half of the studies provided source information for their nesting trait data. Importantly, nesting categories were often inferred from literature observations at the generic or family level, which may obscure important species‐level variation. Finally, definitions for nesting categories were rare. Of the 103 studies that categorized bee species by nesting location, only 25 (24.2%) provided some definition (whether written or by referencing a previous paper's definition) for at least one of their nesting states. We recommend that authors specify the sources of their nesting data, rigorously define the boundaries of their categorizations, and clarify whether data are inferred from the species or generic level.

### 2.3 Diet Breadth

Diet breadth is another trait with important implications for bees' functional roles and responses to environmental change. Because this trait indicates a bee species' range of floral host species, it can determine susceptibility to habitat loss and vulnerability to phenological mismatch. Two‐thirds (100 studies; 65.8%) of the studies in our analysis considered the diet breadth of their focal species (Figure 2d). The majority of these studies classified diet breadth categorically (93 of 100 studies; 93.0%). Most commonly, studies classified bee species as either oligolectic or polylectic, sourcing data from the literature and adhering to the definition that oligolectic species collect pollen from within a single plant family (Table S3). Definitions varied, however, and fewer than half of studies defined these terms at all, whether through written definitions or citations (43 of 100 studies; 43.0%). Importantly, diet breadth can be conceptualized as a continuous variable with large variation in the degree of specialization (Cane and Sipes 2006; Danforth, Minckley, and Neff 2019). Several studies accounted for the diversity of diet specialization states by additionally including such terms as “monolectic” (collecting pollen from a single plant species) and “mesolectic” (collecting pollen from multiple plant genera within the same few plant families, *sensu* Cane and Sipes 2006); (Hall et al. 2019; Hung et al. 2019, 2021; Moretti et al. 2009; Ricotta and Moretti 2011). A minority of studies accounted for the continuous nature of diet breadth by treating it as a numeric variable (in 7 of 100 studies; 7.0%). Studies varied in their approaches to quantifying diet breadth numerically, whether as simply the number of host plant species (Rader et al. 2014), through network analysis (Raiol et al. 2021), or by diversity metrics that consider the phylogenetic breadth of host plant species (Bartomeus et al. 2018; Campbell et al. 2022; Lichtenberg, Mendenhall, and Brosi 2017). It is important to note that these metrics often depend on detailed visitation data, and can be sensitive to effects of sampling bias (Blüthgen 2010). Providing details on the data source (e.g., pollen load data, expert knowledge, and visitation data) is crucial for promoting data reuse in future studies.

### 2.4 Sociality

Sociality is another predictor of ecological patterns in bees, both because of its role in shaping fitness outcomes and environmental interactions, and because of the sheer diversity of social forms in bees (Michener 1974; Wcislo and Fewell 2017). Many social bees may be more resilient than solitary bees to some forms of environmental change, due to advantages of communication strategies, resource sharing, and social behavioral thermoregulation (Ostwald 2024). Defining the lexicon to describe bee sociality has been ongoing and contentious endeavor (Costa and Fitzgerald 1996; Dew, Tierney, and Schwarz 2016; Richards 2019; Wcislo 2005, 1997). This complexity was reflected in the diversity of methods for classifying social forms in the functional trait studies assessed here (Table S3). A common classification method was to divide bees into “social” and “solitary” species, but studies differed in whether “social” referred to all nonsolitary bees, or only to eusocial bees. Fewer studies explicitly distinguished eusocial bees from noneusocial bees that are not solitary, and these differed widely in their classifications. Further, certain inconsistencies in terminology suggested misunderstanding of these lesser studied, “intermediate” forms of sociality, that is, bees that are neither obligately solitary nor obligately eusocial. Examples of problematic classifications of these bees include categorizing all intermediate forms as “semisocial,” or classifying nest aggregations of solitary bees as “communal/semisocial.” Importantly, many bee species exhibit intraspecific variation in social organization (Michener 1974; Shell and Rehan 2017). Indeed, Michener (1974) and others have argued that social labels are often not applicable at the species level because they obscure this intraspecific variation, which tends to be underestimated (Wcislo 2005, 1997). This issue presents a problem for functional trait studies examining sociality, which are generally comparative at the species level and for which it would be prohibitively challenging to assess social organization at the individual or colony level, due to the observation‐intensive nature of this work. Several studies in our analysis addressed this through the use of unique terms for species known to exhibit social polymorphism (e.g., “multiple,” “variable,” “facultatively social”; Bartomeus et al. 2013, 2018; Davis and Comai 2022; Graham et al. 2021; Jacquemin et al. 2020; Moretti et al. 2009; Ricotta and Moretti 2011).

These considerations emphasize the need to clearly define social terminology in functional trait studies, particularly because social categorizations may differ according to the question of interest (Wcislo 2005, 1997). Nevertheless, only a quarter of the studies that measured sociality as a functional trait defined the social terms they used (25 of 105 studies; 23.8%). Valuable examples for defining social terms can be found in the following studies: Ferrari and Polidori (2022), Jacquemin et al. (2020), Kendall et al. (2022), Kratschmer, Kriechbaum, and Pachinger (2018), Pei et al. (2023), Rollin et al. (2015). Several authors have argued that inconsistency in bee social terminology has presented an obstacle to synthesis in phylogenetics (Dew, Tierney, and Schwarz 2016; Kocher and Paxton 2014; Richards 2019); the same is likely to be true in comparative bee functional ecology in the absence of clearly and consistently defined social terms.

### 2.5 Other Traits

Beyond the four most commonly assessed functional traits, several others warrant methodological consideration. Parasitism status, that is, whether or not a given species is a brood parasite or social parasite, was commonly but inconsistently classified, and often excluded from analyses. Studies varied considerably in whether they classified brood parasitism (a reproductive strategy) as a trait state under sociality, nesting biology, diet breadth, multiple of these traits, or as its own trait (e.g., parasite: yes or no). Indeed, parasites may be considered functionally distinct from nonparasitic bees with respect to sociality, nesting, and diet; the appropriate classification scheme will depend in part on the research question. For example, a comparative study assessing impacts of nest microclimate on bee thermal ecology might be better served by assigning parasitic species to the nest type they occupy (e.g., stems), rather than to their own category. Importantly, however, divergence in classification methods across studies will present obstacles to meta‐analysis.

Studies also varied in their approach to measuring tongue (proboscis) length, a functional trait strongly implicated in pollination ecology because it mediates access to diverse floral host species. Tongue length presents measurement challenges because it can require dissection of fresh specimens, a tedious process which can compromise subsequent identification (Cariveau et al. 2016). Likely for this reason, only 13 of the 39 studies measuring tongue length used empirical specimen measurements (Bartomeus et al. 2018; Beyer et al. 2021; Casanelles‐Abella et al. 2023; Eggenberger et al. 2019; Ibanez 2012; Kueneman et al. 2023; Laha et al. 2020; Persson et al. 2015; Ramírez et al. 2015; Ribeiro et al. 2019; Roquer‐Beni et al. 2021; Xie et al. 2023). More commonly, species were categorized as “short” vs. “long” tongued according to the literature, sometimes including an intermediate category (e.g., “medium”). However, this approach obscures important variation within short‐ and long‐tongued groups, and so may not always be appropriate for testing functional hypotheses in pollination ecology. These classifications typically rely only on family information, and so do not capture within‐family variation related to body size. To overcome these limitations, Cariveau et al. (2016) described an allometric equation that explains 91% of the variance in bee tongue length, and produced an R package that allows users to predict tongue length from bee family and ITD (“BeeIT” package). Allometric scaling relationships have been instrumental for developing predictive models to estimate biological measurements, especially in plant ecology (McHale et al. 2009; Montagu et al. 2005; Roxburgh et al. 2015). Since its publication, the BeeIT package was used to estimate tongue length in nine of the functional trait studies we surveyed, suggesting that this approach has reduced quantification barriers (Bartomeus et al. 2018; Beyer et al. 2020; Evans et al. 2018; Hass et al. 2018; Hung et al. 2019; Kratschmer et al. 2021; Lane et al. 2022; Staton et al. 2022; Steinert et al. 2020). This approach will enable analysis of older specimens that cannot be dissected, which will be particularly useful for rare and endangered species. However, caution should be taken when applying this approach broadly, as relationships between body size and tongue length vary across regions and taxa. For example, tongue length varies dramatically in neotropical orchid bees (Apidae: Euglossini), with species of similar body size exhibiting both short and very long tongues, sometimes more than twice their body length. Although overreliance on proxy calculations introduces error that could potentially obscure functional relationships, allometric approaches such as these can represent improvements upon categorical assignments based on bee family alone. However, where possible, empirical tongue measurements are preferable for advancing our documentation and understanding of the functional consequences of tongue length variation.

## 3 Outcomes, Limitations, and Frontiers in Bee Functional Ecology

Functional traits are increasingly providing a popular framework for making generalizable predictions about the impacts of global change on bee biodiversity. The majority of studies analyzed here reported significant effects of environmental variables on functional trait diversity or composition. These findings have helped to clarify patterns of community change in response to environmental disturbances. For example, one generally consistent finding in functional trait studies of urbanization is the tendency for urban environments to favor generalist, cavity‐nesting species (Ayers and Rehan 2021; Banaszak‐Cibicka and Żmihorski 2012; Buchholz and Egerer 2020; Cane et al. 2006; Normandin et al. 2017). In other contexts, however, trait‐mediated responses to environmental change variables may be weak or conflicting across systems (Bartomeus et al. 2018; Greenop, Woodcock, and Pywell 2023; Williams et al. 2010). The extent to which functional trait effects are generalizable across systems appears to be context‐ and trait‐dependent. As a consequence, establishing data standards for trait measurement is crucial for reproducible and reusable data. Below, we provide recommendations for best practices (summarized in Table 1).

### 3.1 Toward Community Data Standards for Bee Trait Data

Meta‐analyses have the potential to clarify patterns in bee functional ecology across biological scales (Bartomeus et al. 2018; Coutinho, Garibaldi, and Viana 2018; Garibaldi et al. 2015; Poulsen and Rasmussen 2020; Woodcock et al. 2019). However, while bee trait data are prolific in the literature, we currently lack community data standards for sharing trait data that would support such meta‐analyses. Trait databases are increasingly emerging as tools for functional exploration within a taxonomic group, with valuable examples from Lepidopteran (Shirey et al. 2022), spider (Pekár et al. 2021), amphibian (Oliveira et al. 2017), plant (Kattge et al. 2011), and bird databases (Tobias et al. 2022); (with many other examples registered in the Open Traits Network; Gallagher et al. 2020). Progress toward aggregated bee trait data will depend on researchers adhering to principles of FAIR (Findable, Accessible, Interoperable, Reusable) data (Wilkinson et al. 2016). Two‐thirds (65.1%) of studies in our review made their trait data available online.

Equally important for making trait data usable in the future analyses is clearly describing trait measurement methods, defining trait terms, and providing comprehensive sampling and specimen data. Where appropriate, researchers should consider adhering to prevailing measurement protocols (Moretti et al. 2017). For example, measuring body size as ITD can help ensure compatibility of data with past and future studies, due to the ubiquity of this measurement method. Importantly, even when using standardized methodologies, methods should still be defined and/or cited to enable future use of data. Importantly, trait data should be shared as raw data to facilitate use in the future analysis. Many datasets in our analysis aggregated trait data at the species level, such that information on within‐species variation was lost. Additionally, raw numeric trait data should be shared even if data were binned into categories for analysis (i.e., share individual ITD measurements even if data were binned as “small” vs. “large” in analyses). Associated geographic and taxonomic data should likewise adhere to community data standards (e.g., Darwin Core). In our review, we found that taxonomic information was at times incomplete, inaccurate, or ambiguous, and geographic data were poorly linked to specimen‐level trait data and/or formatted according to outdated standards (Degrees, Minutes, Seconds format). To resolve ambiguity and promote machine‐readability across datasets, taxonomic information should be linked to taxonomic identifiers (e.g., GBIF Backbone Taxonomy 2023) and sampling coordinates should be reported in decimal‐degree format.

Functional traits that are typically represented categorically (especially behavioral traits, e.g., nesting biology, sociality, and diet breadth) present unique challenges for data harmonization. Traits typically measured numerically (e.g., body size) can be measured consistently across species, allowing for interspecific comparisons at all spatial scales. Most behavioral traits, by contrast, rely on prior categorizations sourced from the literature and often rely on data that is geographically limited, potentially obscuring important interspecific variation. Additionally, we currently lack a controlled vocabulary for bee trait classifiers (see examples from other taxa, Gkoutos et al. 2015), which explains the wide diversity of adopted trait terminology observed in our study (Table S3). In the absence of a controlled vocabulary, terminology should be clearly defined, whether by written definitions or citations of existing definitions, including links to ontologies, wherever possible (e.g., the Hymenoptera Anatomy Ontology; Yoder et al. 2010).

As a template for standardized bee trait data sharing, we have compiled and harmonized the primary morphological data presented in the studies we reviewed, where data were available and interpretable, available at https://zenodo.org/doi/10.5281/zenodo.10139286 as Table S4 and registered in the Open Traits Network (Gallagher et al. 2020); (Blank template: Table S5). This dataset presents nearly 12,000 records of body size, tongue size, and pilosity measurements for 1622 bee species. Behavioral trait data (e.g., nesting biology, sociality, and diet breadth) in the studies we analyzed were almost always extracted from the literature (secondary data), and so do not feature in this primary dataset. We harmonized data by manually interpreting datasets and classifying data values by trait (e.g., body size and tongue size), measurement type (e.g., ITD and wing length), record level (whether data were provided at the specimen level or summarized at the species level), and other features explained in the metadata. Where possible, data classes are mapped to the Darwin Core standard, a widely used glossary of terms for biodiversity data sharing (Wieczorek et al. 2012). We also present sampling data (e.g., geographic coordinates, collection date, habitat type, and life stage), for each entry, where available. To resolve ambiguity around presentation of taxonomic names, we have mapped the presented taxon names to standardized taxonomic identifiers so that names are provided in both machine‐readable and human‐readable formats (e.g., “X.SONORINA,” “*Xylocopa sonorina* Smith, 1874” and “Xylocopa varipuncta” [synonym] all resolve to “gbif:9016167”); (GBIF Backbone Taxonomy 2023). Finally, to resolve ambiguity around trait measurement methods, we have introduced new trait definitions to the Hymenoptera Anatomy Ontology (Yoder et al. 2010) to link functional trait data to unique, persistent identifiers (e.g., tongue length: https://purl.obolibrary.org/obo/HAO_0002606). This approach facilitates data reuse by allowing researchers to indicate unambiguously when measurement protocols conform to particular standards (e.g., tongue length measured as the combined length of the prementum and glossa).

### 3.2 What Are the Gaps in Our Understanding of Bee Functional Ecology?

Our analysis revealed critical knowledge gaps in the field of comparative bee functional ecology. While a subset of morphological and behavioral traits was well‐represented, phenological traits were more rare and physiological trait data was nearly absent. Despite a wealth of physiological research on honey bees and bumble bees, physiological trait data for other bee species has lagged. In other insect taxa, comparative physiological trait data (e.g., thermal tolerance and desiccation resistance) have been usefully leveraged to understand and predict performance under climate change (Baudier et al. 2015; Bujan, Yanoviak, and Kaspari 2016; Bujan et al. 2019; Roeder et al. 2021). Interest in quantifying these traits in nonhoney bees is increasing (Burdine and McCluney 2019; Gonzalez et al. 2020; da Silva et al. 2021), yet they are still rare in comparative functional trait studies (Hamblin et al. 2017), due perhaps to the labor‐intensive nature of quantifying these traits, especially relative to better‐studied traits that can be sourced from the literature. Prioritizing physiological trait data collection and data sharing will vastly expand opportunities to predict performance under future climate scenarios. Phenological traits, especially flight seasonality, were poorly represented in the studies we analyzed. These traits may influence susceptibility to environmental change, and merit increased attention in future functional trait studies. While we emphasize the need to fill these trait gaps, we equally stress that trait selection should generally be hypothesis driven.

Urgently, future work should also expand the topical and geographic breadth of functional trait studies. The vast majority of functional trait studies have been conducted in Europe and North America (76.3% of studies), mirroring a larger bias in ecological research (Archer et al. 2014; Martin, Blossey, and Ellis 2012; Pyšek et al. 2008). The geographic bias in bee functional trait research is even more extreme than the one reported by Winfree, Bartomeus, and Cariveau (2011) for studies of native pollinators in human‐altered landscapes (52% of studies conducted in Europe and North America). Preserving global crop pollination is a top priority for sustaining food security, yet relevant data are concentrated in wealthy regions that are lowest priority for this aim. Overreliance on geographically restricted data will undercut our ability to predict bee responses to environmental change globally. Finally, these studies were dominated by research on agroecosystems and agricultural/land use questions. This contrasts with a broader pattern in terrestrial ecology, where natural systems tend to be overrepresented in ecological studies (Martin, Blossey, and Ellis 2012). Climate change questions, in particular, were poorly represented in the studies analyzed here. Increasing attention to these topical gaps will help balance the body of functional trait literature better in line with conservation priorities.

## 4 Synthesis and Concluding Remarks

Variation in functional traits significantly predicts patterns of community change across a wide range of systems and contexts. Our review not only highlights common approaches to morphological trait measurement (e.g., ITD) but also reveals knowledge gaps in bee trait data and terminological inconsistency in classifiers applied to behavioral traits, namely diet breadth, nesting behavior, and sociality. We do not prescribe a particular terminology structure here, but rather emphasize that when authors clearly define terms their data becomes useful beyond its original publication. Our analysis highlights the need for integration of data standards and open science principles into bee functional ecology research. To promote data reuse, researchers should rigorously define trait terminology and make trait data openly accessible with clear metadata and methodological descriptions. Our template for bee functional trait data sharing, along with the compiled primary data from these studies, represents an initial step toward a consolidated database of bee functional traits. Future work toward this aim will promote synthesis across diverse study systems and questions in bee functional ecology.

## Author Contributions

**Madeleine M. Ostwald:** conceptualization (equal), data curation (equal), formal analysis (lead), writing – original draft (lead), writing – review and editing (lead). **Victor H. Gonzalez:** conceptualization (equal), data curation (equal), writing – original draft (supporting), writing – review and editing (equal). **Carrie Chang:** data curation (equal). **Nydia Vitale:** data curation (equal), writing – review and editing (supporting). **Mariano Lucia:** data curation (equal), writing – review and editing (supporting). **Katja C. Seltmann:** conceptualization (equal), funding acquisition (lead), writing – review and editing (equal).

## Conflicts of Interest

The authors declare no conflicts of interest.

## Supporting information


**Table S1.** Summarized information on functional traits commonly measured in bee ecological studies, with their functional significance, common methods of measurement in bee studies, and common states for traits involving categorical data.

**Table S2.** Metadata from 152 studies analyzed in our review, including citations, the traits analyzed, the types of trait measurements, and the data sources.

**Table S3.** Trait classes, definitions, and associated data sources, where available, for the three most commonly studied behavioral traits: nesting biology, sociality, and diet breadth.

**Table S4.** Harmonized primary morphological data compiled from our focal studies (see “Guide to the Morphological Dataset and the Data Upload Template” for metadata).

**Table S5.** Template for standardized presentation of trait data. Column headers described below (see “Guide to the Morphological Dataset and the Data Upload Template”).

**Data S1.** Data source.

## Data Availability

All data associated with the manuscript is publicly available at https://zenodo.org/doi/10.5281/zenodo.10139286.
