# Supplementary material for: Toward a Functional Trait Approach to Bee Ecology
Source: Ecol Evol. 2024 Oct 18;14(10):e70465. doi: 10.1002/ece3.70465 (PMC11487340; doi:10.1002/ece3.70465)
Supplement: Supplementary file 6 — Data S1. Data source. [file ECE3-14-e70465-s002.docx]

**Data Sources**

Aguirre-Gutiérrez, J., Kissling, W. D., Carvalheiro, L. G., WallisDeVries, M. F., Franzén, M., & Biesmeijer, J. C. (2016). Functional traits help to explain half-century long shifts in pollinator distributions. *Scientific Reports*, *6*(1), 24451. <https://doi.org/10.1038/srep24451>

Araújo, E. D., Costa, M., Chaud-Netto, J., & Fowler, H. G. (2004). Body size and flight distance in stingless bees (Hymenoptera: Meliponini): inference of flight range and possible ecological implications. *Brazilian Journal of Biology*, *64*(3b), 563–568. <https://doi.org/10.1590/S1519-69842004000400003>

Banaszak-Cibicka, W., & Dylewski, Ł. (2021). Species and functional diversity—A better understanding of the impact of urbanization on bee communities. *Science of The Total Environment*, *774*, 145729. <https://doi.org/10.1016/j.scitotenv.2021.145729>

Banaszak-Cibicka, W., & Żmihorski, M. (2012). Wild bees along an urban gradient: Winners and losers. *Journal of Insect Conservation*, *16*(3), 331–343. <https://doi.org/10.1007/s10841-011-9419-2>

Bartomeus, I., Ascher, J. S., Gibbs, J., Danforth, B. N., Wagner, D. L., Hedtke, S. M., & Winfree, R. (2013). Historical changes in northeastern US bee pollinators related to shared ecological traits. *Proceedings of the National Academy of Sciences*, *110*(12), 4656–4660. <https://doi.org/10.1073/pnas.1218503110>

Bartomeus, I., Cariveau, D. P., Harrison, T., & Winfree, R. (2018). On the inconsistency of pollinator species traits for predicting either response to land-use change or functional contribution. *Oikos*, *127*(2), 306–315. <https://doi.org/10.1111/oik.04507>

Beyer, N., Gabriel, D., Kirsch, F., Schulz‐Kesting, K., Dauber, J., & Westphal, C. (2020). Functional groups of wild bees respond differently to faba bean *Vicia faba* L. cultivation at landscape scale. *Journal of Applied Ecology*, *57*(12), 2499–2508. <https://doi.org/10.1111/1365-2664.13745>

Beyer, N., Kirsch, F., Gabriel, D., & Westphal, C. (2021). Identity of mass-flowering crops moderates functional trait composition of pollinator communities. *Landscape Ecology*, *36*, 2657–2671. <https://doi.org/10.1007/s10980-021-01261-3>

Bommarco, R., Biesmeijer, J. C., Meyer, B., Potts, S. G., Pöyry, J., Roberts, S. P. M., Steffan-Dewenter, I., & Öckinger, E. (2010). Dispersal capacity and diet breadth modify the response of wild bees to habitat loss. *Proceedings of the Royal Society B: Biological Sciences*, *277*(1690), 2075–2082. <https://doi.org/10.1098/rspb.2009.2221>

Braman, C. A., McCarty, E., Ulyshen, M. D., Janvier, A. J., Traylor, C., Edelkind-Vealey, M., & Braman, S. K. (2023). Urban bee functional groups response to landscape context in the Southeastern US. *Frontiers in Sustainable Cities*, *5*, 1192588. <https://doi.org/10.3389/frsc.2023.1192588>

Brasil, S. N. R., George, M., & Rehan, S. M. (2024). Functional traits of wild bees in response to urbanization. *Journal of Insect Conservation*, *28*(1), 127–139. <https://doi.org/10.1007/s10841-023-00528-1>

Buchholz, S., Gathof, A. K., Grossmann, A. J., Kowarik, I., & Fischer, L. K. (2020). Wild bees in urban grasslands: Urbanisation, functional diversity and species traits. *Landscape and Urban Planning*, *196*, 103731. <https://doi.org/10.1016/j.landurbplan.2019.103731>

Cabral Borges, R., Padovani, K., Imperatriz-Fonseca, V. L., & Giannini, T. C. (2020). A dataset of multi-functional ecological traits of Brazilian bees. *Scientific Data*, *7*(1), 120. <https://doi.org/10.1038/s41597-020-0461-3>

Campbell, A. J., Lichtenberg, E. M., Carvalheiro, L. G., Menezes, C., Borges, R. C., Coelho, B. W. T., Freitas, M. A. B., Giannini, T. C., Leão, K. L., de Oliveira, F. F., Silva, T. S. F., & Maués, M. M. (2022). High bee functional diversity buffers crop pollination services against Amazon deforestation. *Agriculture, Ecosystems & Environment*, *326*, 107777. <https://doi.org/10.1016/j.agee.2021.107777>

Cane, J. H., Minckley, R. L., Kervin, L. J., Roulston, T. H., & Williams, N. M. (2006). Complex Responses Within A Desert Bee Guild (Hymenoptera: Apiformes) To Urban Habitat Fragmentation. *Ecological Applications*, *16*(2), 632–644. [https://doi.org/10.1890/1051-0761(2006)016[0632:CRWADB]2.0.CO;2](https://doi.org/10.1890/1051-0761(2006)016%5b0632:CRWADB%5d2.0.CO;2)

Carper, A. L., Adler, L. S., Warren, P. S., & Irwin, R. E. (2014). Effects of Suburbanization on Forest Bee Communities. *Environmental Entomology*, *43*(2), 253–262. <https://doi.org/10.1603/EN13078>

Carrié, R., Andrieu, E., Cunningham, S. A., Lentini, P. E., Loreau, M., & Ouin, A. (2017). Relationships among ecological traits of wild bee communities along gradients of habitat amount and fragmentation. *Ecography*, *40*(1), 85–97. <https://doi.org/10.1111/ecog.02632>

Casanelles-Abella, J., Fontana, S., Fournier, B., Frey, D., & Moretti, M. (2023). Low resource availability drives feeding niche partitioning between wild bees and honeybees in a European city. *Ecological Applications*, *33*(1), e2727. <https://doi.org/10.1002/eap.2727>

Casanelles-Abella, J., Fontana, S., Meier, E., Moretti, M., & Fournier, B. (2023). Spatial mismatch between wild bee diversity hotspots and protected areas. *Conservation Biology*, *37*(4), e14082. <https://doi.org/10.1111/cobi.14082>

Cecala, J. M., & Wilson Rankin, E. E. (2021). Wild bee functional diversity and plant associations in native and conventional plant nurseries. *Ecological Entomology*, *46*(6), 1283–1292. <https://doi.org/10.1111/een.13074>

Chase, M. H., Charles, B., Harmon‐Threatt, A., & Fraterrigo, J. M. (2023). Diverse forest management strategies support functionally and temporally distinct bee communities. *Journal of Applied Ecology*, *60*(11), 2375–2388. <https://doi.org/10.1111/1365-2664.14513>

Cohen, H., Egerer, M., Thomas, S.-S., & Philpott, S. M. (2022). Local and landscape features constrain the trait and taxonomic diversity of urban bees. *Landscape Ecology*, *37*(2), 583–599. <https://doi.org/10.1007/s10980-021-01370-z>

Cortés-Gómez, A. M., González-Chaves, A., Urbina-Cardona, N., & Garibaldi, L. A. (2023). Functional Traits in Bees: The Role of Body Size and Hairs in the Pollination of a Passiflora Crop. *Neotropical Entomology*, *52*(4), 642–651. <https://doi.org/10.1007/s13744-023-01058-w>

Coutinho, J. G. D. E., Garibaldi, L. A., & Viana, B. F. (2018). The influence of local and landscape scale on single response traits in bees: A meta-analysis. *Agriculture, Ecosystems & Environment*, *256*, 61–73. <https://doi.org/10.1016/j.agee.2017.12.025>

Coutinho, J. G. da E., Garibaldi, L. A., & Viana, B. F. (2018). The influence of local and landscape scale on single response traits in bees: A meta-analysis. *Agriculture, Ecosystems & Environment*, *256*, 61–73. <https://doi.org/10.1016/j.agee.2017.12.025>

Coutinho, J. G. E., Hipólito, J., Santos, R. L. S., Moreira, E. F., Boscolo, D., & Viana, B. F. (2021). Landscape Structure Is a Major Driver of Bee Functional Diversity in Crops. *Frontiers in Ecology and Evolution*, *9*, 624835. <https://doi.org/10.3389/fevo.2021.624835>

Cunningham‐Minnick, M. J., Peters, V. E., & Crist, T. O. (2020). Bee communities and pollination services in adjacent crop fields following flower removal in an invasive forest shrub. *Ecological Applications*, *30*(4), e02078. <https://doi.org/10.1002/eap.2078>

Dalmazzo, M. (2010). *Diversidad y aspectos biológicos de abejas silvestres de un ambiente urbano y otro natural de la región central de Santa Fe, Argentina*.

Dalmazzo, M., Zumoffen, L., Ghiglione, C., Roig-Alsina, A., & Chacoff, N. (2024). Diversity and biological traits of bees visiting flowers of Cucurbita maxima var. Zapallito differ between biodiversity-based and conventional management practices. *Environmental Monitoring and Assessment*, *196*(1), 6. <https://doi.org/10.1007/s10661-023-12161-1>

Davies, C. B., & Davis, T. S. (2023). Social but not solitary bee abundance tracks pollen protein accumulation in forest canopy gaps. *Ecological Entomology*, *48*(6), 738–754. <https://doi.org/10.1111/een.13269>

Davis, T. S., & Comai, N. (2022). Canopy cover and seasonality are associated with variation in native bee assemblages across a mixed pine‐juniper woodland. *Agricultural and Forest Entomology*, *24*(4), 497–505. <https://doi.org/10.1111/afe.12511>

de Bello, F., Lepš, J., Lavorel, S., & Moretti, M. (2007). Importance of species abundance for assessment of trait composition: An example based on pollinator communities. *Community Ecology*, *8*(2), 163–170. <https://doi.org/10.1556/ComEc.8.2007.2.3>

De Palma, A., Kuhlmann, M., Roberts, S. P. M., Potts, S. G., Börger, L., Hudson, L. N., Lysenko, I., Newbold, T., & Purvis, A. (2015). Ecological traits affect the sensitivity of bees to land‐use pressures in E uropean agricultural landscapes. *Journal of Applied Ecology*, *52*(6), 1567–1577. <https://doi.org/10.1111/1365-2664.12524>

Dorian, N. N., McCarthy, M. W., & Crone, E. E. (2022). Ecological traits explain long‐term phenological trends in solitary bees. *Journal of Animal Ecology*, 1365-2656.13778. <https://doi.org/10.1111/1365-2656.13778>

Edelkind-Vealey, M., Ulyshen, M. D., & Braman, S. K. (2024). Local factors influence the wild bee functional community at the urban-forest interface. *Frontiers in Ecology and Evolution*, *12*, 1389619. <https://doi.org/10.3389/fevo.2024.1389619>

Eggenberger, H., Frey, D., Pellissier, L., Ghazoul, J., Fontana, S., & Moretti, M. (2019). Urban bumblebees are smaller and more phenotypically diverse than their rural counterparts. *Journal of Animal Ecology*, *88*(10), 1522–1533. <https://doi.org/10.1111/1365-2656.13051>

Ekroos, J., Rundlöf, M., & Smith, H. G. (2013). Trait-dependent responses of flower-visiting insects to distance to semi-natural grasslands and landscape heterogeneity. *Landscape Ecology*, *28*(7), 1283–1292. <https://doi.org/10.1007/s10980-013-9864-2>

Evans, E., Smart, M., Cariveau, D., & Spivak, M. (2018). Wild, native bees and managed honey bees benefit from similar agricultural land uses. *Agriculture, Ecosystems & Environment*, *268*, 162–170. <https://doi.org/10.1016/j.agee.2018.09.014>

Everaars, J., Settele, J., & Dormann, C. F. (2018). Fragmentation of nest and foraging habitat affects time budgets of solitary bees, their fitness and pollination services, depending on traits: Results from an individual-based model. *PLOS ONE*, *13*(2), e0188269. <https://doi.org/10.1371/journal.pone.0188269>

Fauviau, A., Baude, M., Bazin, N., Fiordaliso, W., Fisogni, A., Fortel, L., Garrigue, J., Geslin, B., Goulnik, J., Guilbaud, L., Hautekèete, N., Heiniger, C., Kuhlmann, M., Lambert, O., Langlois, D., Le Féon, V., Lopez Vaamonde, C., Maillet, G., Massol, F., … Henry, M. (2022). A large-scale dataset reveals taxonomic and functional specificities of wild bee communities in urban habitats of Western Europe. *Scientific Reports*, *12*(1), 18866. <https://doi.org/10.1038/s41598-022-21512-w>

Felderhoff, J., Gathof, A. K., Buchholz, S., & Egerer, M. (2023). Vegetation complexity and nesting resource availability predict bee diversity and functional traits in community gardens. *Ecological Applications*, *33*(2), e2759. <https://doi.org/10.1002/eap.2759>

Ferrari, A., & Polidori, C. (2022). How city traits affect taxonomic and functional diversity of urban wild bee communities: Insights from a worldwide analysis. *Apidologie*, *53*(4), 46. <https://doi.org/10.1007/s13592-022-00950-5>

Figueroa, L. L., Compton, S., Grab, H., & McArt, S. H. (2021). Functional traits linked to pathogen prevalence in wild bee communities. *Scientific Reports*, *11*, 7529. <https://doi.org/10.1038/s41598-021-87103-3>

Forrest, J. R. K., Thorp, R. W., Kremen, C., & Williams, N. M. (2015). Contrasting patterns in species and functional-trait diversity of bees in an agricultural landscape. *Journal of Applied Ecology*, *52*(3), 706–715. <https://doi.org/10.1111/1365-2664.12433>

Fortel, L., Henry, M., Guilbaud, L., Guirao, A. L., Kuhlmann, M., Mouret, H., Rollin, O., & Vaissière, B. E. (2014). Decreasing Abundance, Increasing Diversity and Changing Structure of the Wild Bee Community (Hymenoptera: Anthophila) along an Urbanization Gradient. *PLoS ONE*, *9*(8), e104679. <https://doi.org/10.1371/journal.pone.0104679>

Fortuin, C. C., & Gandhi, K. J. K. (2021). Functional traits and nesting habitats distinguish the structure of bee communities in clearcut and managed hardwood & pine forests in Southeastern USA. *Forest Ecology and Management*, *496*, 119351. <https://doi.org/10.1016/j.foreco.2021.119351>

Garibaldi, L. A., Bartomeus, I., Bommarco, R., Klein, A. M., Cunningham, S. A., Aizen, M. A., Boreux, V., Garratt, M. P. D., Carvalheiro, L. G., Kremen, C., Morales, C. L., Schüepp, C., Chacoff, N. P., Freitas, B. M., Gagic, V., Holzschuh, A., Klatt, B. K., Krewenka, K. M., Krishnan, S., … Woyciechowski, M. (2015). Trait matching of flower visitors and crops predicts fruit set better than trait diversity. *Journal of Applied Ecology*, *52*(6), 1436–1444. <https://doi.org/10.1111/1365-2664.12530>

Geppert, C., Cappellari, A., Corcos, D., Caruso, V., Cerretti, P., Mei, M., & Marini, L. (2023). Temperature and not landscape composition shapes wild bee communities in an urban environment. *Insect Conservation and Diversity*, *16*(1), 65–76. <https://doi.org/10.1111/icad.12602>

Giannini, T. C., Costa, W. F., Borges, R. C., Miranda, L., da Costa, C. P. W., Saraiva, A. M., & Imperatriz Fonseca, V. L. (2020). Climate change in the Eastern Amazon: Crop-pollinator and occurrence-restricted bees are potentially more affected. *Regional Environmental Change*, *20*, 9. <https://doi.org/10.1007/s10113-020-01611-y>

Glenny, W., Runyon, J. B., & Burkle, L. A. (2023). Habitat characteristics structuring bee communities in a forest-shrubland ecotone. *Forest Ecology and Management*, *534*, 120883. <https://doi.org/10.1016/j.foreco.2023.120883>

Grab, H., Branstetter, M. G., Amon, N., Urban-Mead, K. R., Park, M. G., Gibbs, J., Blitzer, E. J., Poveda, K., Loeb, G., & Danforth, B. N. (2019). Agriculturally dominated landscapes reduce bee phylogenetic diversity and pollination services. *Science*, *363*(6424), 282–284. <https://doi.org/10.1126/science.aat6016>

Graf, L. V., Schneiberg, I., & Gonçalves, R. B. (2022a). Bee functional groups respond to vegetation cover and landscape diversity in a Brazilian metropolis. *Landscape Ecology*, *37*(4), 1075–1089. <https://doi.org/10.1007/s10980-022-01430-y>

Graf, L. V., Schneiberg, I., & Gonçalves, R. B. (2022b). Bee functional groups respond to vegetation cover and landscape diversity in a Brazilian metropolis. *Landscape Ecology*, *37*(4), 1075–1089. <https://doi.org/10.1007/s10980-022-01430-y>

Graham, K. K., Gibbs, J., Wilson, J., May, E., & Isaacs, R. (2021). Resampling of wild bees across fifteen years reveals variable species declines and recoveries after extreme weather. *Agriculture, Ecosystems & Environment*, *317*, 107470. <https://doi.org/10.1016/j.agee.2021.107470>

Greenleaf, S. S., Williams, N. M., Winfree, R., & Kremen, C. (2007). Bee foraging ranges and their relationship to body size. *Oecologia*, *153*(3), 589–596. <https://doi.org/10.1007/s00442-007-0752-9>

Gruver, A., & CaraDonna, P. (2021). Chicago Bees: Urban Areas Support Diverse Bee Communities but With More Non-Native Bee Species Compared to Suburban Areas. *Environmental Entomology*, *50*(4), 982–994. <https://doi.org/10.1093/ee/nvab048>

Guenat, S., Kunin, W. E., Dougill, A. J., & Dallimer, M. (2019). Effects of urbanisation and management practices on pollinators in tropical Africa. *Journal of Applied Ecology*, *56*(1), 214–224. <https://doi.org/10.1111/1365-2664.13270>

Gutiérrez-Chacón, C., Valderrama-A, C., & Klein, A.-M. (2020). Biological corridors as important habitat structures for maintaining bees in a tropical fragmented landscape. *Journal of Insect Conservation*, *24*(1), 187–197. <https://doi.org/10.1007/s10841-019-00205-2>

Hahs, A. K., Fournier, B., Aronson, M. F. J., Nilon, C. H., Herrera-Montes, A., Salisbury, A. B., Threlfall, C. G., Rega-Brodsky, C. C., Lepczyk, C. A., La Sorte, F. A., MacGregor-Fors, I., Scott MacIvor, J., Jung, K., Piana, M. R., Williams, N. S. G., Knapp, S., Vergnes, A., Acevedo, A. A., Gainsbury, A. M., … Moretti, M. (2023). Urbanisation generates multiple trait syndromes for terrestrial animal taxa worldwide. *Nature Communications*, *14*(1), 4751. <https://doi.org/10.1038/s41467-023-39746-1>

Hall, M. A., Nimmo, D. G., Cunningham, S. A., Walker, K., & Bennett, A. F. (2019). The response of wild bees to tree cover and rural land use is mediated by species’ traits. *Biological Conservation*, *231*, 1–12. <https://doi.org/10.1016/j.biocon.2018.12.032>

Hamblin, A. L., Youngsteadt, E., & Frank, S. D. (2018). Wild bee abundance declines with urban warming, regardless of floral density. *Urban Ecosystems*, *21*(3), 419–428. <https://doi.org/10.1007/s11252-018-0731-4>

Hamblin, A. L., Youngsteadt, E., Lopez-Uribe, M. M., & Frank, S. D. (2017). Physiological thermal limits predict differential responses of bees to urban heat-island effects. *Biology Letters*, *13*, 0125.

Harmon-Threatt, A. N., & Anderson, N. L. (2023). Bee movement between natural fragments is rare despite differences in species, patch, and matrix variables. *Landscape Ecology*, *38*(10), 2519–2531. <https://doi.org/10.1007/s10980-023-01719-6>

Harrison, T., Gibbs, J., & Winfree, R. (2018). Forest bees are replaced in agricultural and urban landscapes by native species with different phenologies and life-history traits. *Global Change Biology*, *24*(1), 287–296. <https://doi.org/10.1111/gcb.13921>

Hass, A. L., Liese, B., Heong, K. L., Settele, J., Tscharntke, T., & Westphal, C. (2018). Plant-pollinator interactions and bee functional diversity are driven by agroforests in rice-dominated landscapes. *Agriculture, Ecosystems & Environment*, *253*, 140–147. <https://doi.org/10.1016/j.agee.2017.10.019>

Hevia, V., Carmona, C. P., Azcárate, F. M., Heredia, R., & González, J. A. (2021). Role of floral strips and semi-natural habitats as enhancers of wild bee functional diversity in intensive agricultural landscapes. *Agriculture, Ecosystems & Environment*, *319*, 107544. <https://doi.org/10.1016/j.agee.2021.107544>

Hoiss, B., Krauss, J., Potts, S. G., Roberts, S., & Steffan-Dewenter, I. (2012a). Altitude acts as an environmental filter on phylogenetic composition, traits and diversity in bee communities. *Proceedings of the Royal Society B: Biological Sciences*, *279*(1746), 4447–4456. <https://doi.org/10.1098/rspb.2012.1581>

Hoiss, B., Krauss, J., Potts, S. G., Roberts, S., & Steffan-Dewenter, I. (2012b). Altitude acts as an environmental filter on phylogenetic composition, traits and diversity in bee communities. *Proceedings of the Royal Society B: Biological Sciences*, *279*(1746), 4447–4456. <https://doi.org/10.1098/rspb.2012.1581>

Hopfenmüller, S., Steffan-Dewenter, I., & Holzschuh, A. (2014). Trait-Specific Responses of Wild Bee Communities to Landscape Composition, Configuration and Local Factors. *PLoS ONE*, *9*(8), e104439. <https://doi.org/10.1371/journal.pone.0104439>

Hung, K. J., Ascher, J. S., Davids, J. A., & Holway, D. A. (2019). Ecological filtering in scrub fragments restructures the taxonomic and functional composition of native bee assemblages. *Ecology*, *100*(5). <https://doi.org/10.1002/ecy.2654>

Hung, K.-L. J., Sandoval, S. S., Ascher, J. S., & Holway, D. A. (2021). Joint Impacts of Drought and Habitat Fragmentation on Native Bee Assemblages in a California Biodiversity Hotspot. *Insects*, *12*(2), 135. <https://doi.org/10.3390/insects12020135>

Ibanez, S. (2012). Optimizing size thresholds in a plant–pollinator interaction web: Towards a mechanistic understanding of ecological networks. *Oecologia*, *170*(1), 233–242. <https://doi.org/10.1007/s00442-012-2290-3>

Jacobs, J., Beenaerts, N., & Artois, T. (2023). Green roofs and pollinators, useful green spots for some wild bee species (Hymenoptera: Anthophila), but not so much for hoverflies (Diptera: Syrphidae). *Scientific Reports*, *13*(1), 1449. <https://doi.org/10.1038/s41598-023-28698-7>

Jacquemin, F., Violle, C., Munoz, F., Mahy, G., Rasmont, P., Roberts, S. P. M., Vray, S., & Dufrêne, M. (2020). Loss of pollinator specialization revealed by historical opportunistic data: Insights from network-based analysis. *PLOS ONE*, *15*(7), e0235890. <https://doi.org/10.1371/journal.pone.0235890>

Jauker, B., Krauss, J., Jauker, F., & Steffan-Dewenter, I. (2013). Linking life history traits to pollinator loss in fragmented calcareous grasslands. *Landscape Ecology*, *28*(1), 107–120. <https://doi.org/10.1007/s10980-012-9820-6>

Kammerer, M., Goslee, S. C., Douglas, M. R., Tooker, J. F., & Grozinger, C. M. (2021). Wild bees as winners and losers: Relative impacts of landscape composition, quality, and climate. *Global Change Biology*, *27*(6), 1250–1265. <https://doi.org/10.1111/gcb.15485>

Kazenel, M. R., Wright, K. W., Griswold, T., Whitney, K. D., & Rudgers, J. A. (2024). Heat and desiccation tolerances predict bee abundance under climate change. *Nature*, *628*(8007), 342–348. <https://doi.org/10.1038/s41586-024-07241-2>

Kendall, L. K., Mola, J. M., Portman, Z. M., Cariveau, D. P., Smith, H. G., & Bartomeus, I. (2022). The potential and realized foraging movements of bees are differentially determined by body size and sociality. *Ecology*, *103*(11), e3809. <https://doi.org/10.1002/ecy.3809>

Kratschmer, S., Kriechbaum, M., & Pachinger, B. (2018). Buzzing on top: Linking wild bee diversity, abundance and traits with green roof qualities. *Urban Ecosystems*, *21*(3), 429–446. <https://doi.org/10.1007/s11252-017-0726-6>

Kratschmer, S., Pachinger, B., Gaigher, R., Pryke, J. S., van Schalkwyk, J., Samways, M. J., Melin, A., Kehinde, T., Zaller, J. G., & Winter, S. (2021). Enhancing flowering plant functional richness improves wild bee diversity in vineyard inter‐rows in different floral kingdoms. *Ecology and Evolution*, *11*(12), 7927–7945. <https://doi.org/10.1002/ece3.7623>

Kratschmer, S., Pachinger, B., Schwantzer, M., Paredes, D., Guernion, M., Burel, F., Nicolai, A., Strauss, P., Bauer, T., Kriechbaum, M., Zaller, J. G., & Winter, S. (2018). Tillage intensity or landscape features: What matters most for wild bee diversity in vineyards? *Agriculture, Ecosystems & Environment*, *266*, 142–152. <https://doi.org/10.1016/j.agee.2018.07.018>

Kratschmer, S., Pachinger, B., Schwantzer, M., Paredes, D., Guzmán, G., Goméz, J. A., Entrenas, J. A., Guernion, M., Burel, F., Nicolai, A., Fertil, A., Popescu, D., Macavei, L., Hoble, A., Bunea, C., Kriechbaum, M., Zaller, J. G., & Winter, S. (2019). Response of wild bee diversity, abundance, and functional traits to vineyard inter‐row management intensity and landscape diversity across Europe. *Ecology and Evolution*, *9*(7), 4103–4115. <https://doi.org/10.1002/ece3.5039>

Kueneman, J. G., Bonadies, E., Thomas, D., Roubik, D. W., & Wcislo, W. T. (2023). Neotropical bee microbiomes point to a fragmented social core and strong species-level effects. *Microbiome*, *11*(1), 150. <https://doi.org/10.1186/s40168-023-01593-z>

Laha, S., Chatterjee, S., Das, A., Smith, B., & Basu, P. (2020). Exploring the importance of floral resources and functional trait compatibility for maintaining bee fauna in tropical agricultural landscapes. *Journal of Insect Conservation*, *24*(3), 431–443. <https://doi.org/10.1007/s10841-020-00225-3>

Lane, I. G., Portman, Z. M., Herron‐Sweet, C. H., Pardee, G. L., & Cariveau, D. P. (2021). Differences in bee community composition between restored and remnant prairies are more strongly linked to forb community differences than landscape differences. *Journal of Applied Ecology*, *59*(1), 129–140. <https://doi.org/10.1111/1365-2664.14035>

Lazarina, M., Sgardelis, S. P., Tscheulin, T., Kallimanis, A. S., Devalez, J., & Petanidou, T. (2016). Bee response to fire regimes in Mediterranean pine forests: The role of nesting preference, trophic specialization, and body size. *Basic and Applied Ecology*, *17*(4), 308–320. <https://doi.org/10.1016/j.baae.2016.02.001>

Le Féon, V., Poggio, S. L., Torretta, J. P., Bertrand, C., Molina, G. A. R., Burel, F., Baudry, J., & Ghersa, C. M. (2016). Diversity and life-history traits of wild bees (Insecta: Hymenoptera) in intensive agricultural landscapes in the Rolling Pampa, Argentina. *Journal of Natural History*, *50*(19–20), 1175–1196. <https://doi.org/10.1080/00222933.2015.1113315>

Lichtenberg, E. M., Mendenhall, C. D., & Brosi, B. (2017). Foraging traits modulate stingless bee community disassembly under forest loss. *Journal of Animal Ecology*, *86*(6), 1404–1416. <https://doi.org/10.1111/1365-2656.12747>

Maas, B., Brandl, M., Hussain, R. I., Frank, T., Zulka, K. P., Rabl, D., Walcher, R., & Moser, D. (2021). Functional traits driving pollinator and predator responses to newly established grassland strips in agricultural landscapes. *Journal of Applied Ecology*, *58*(8), 1728–1737. <https://doi.org/10.1111/1365-2664.13892>

MacInnis, G., Normandin, E., & Ziter, C. D. (2023). Decline in wild bee species richness associated with honey bee (Apis mellifera L.) abundance in an urban ecosystem. *PeerJ*, *11*, e14699. <https://doi.org/10.7717/peerj.14699>

Main, A. R., Webb, E. B., Goyne, K. W., & Mengel, D. (2019). Field-level characteristics influence wild bee functional guilds on public lands managed for conservation. *Global Ecology and Conservation*, *17*, e00598. <https://doi.org/10.1016/j.gecco.2019.e00598>

Marcacci, G., Grass, I., Rao, V. S., Kumar S, S., Tharini, K. B., Belavadi, V. V., Nölke, N., Tscharntke, T., & Westphal, C. (2022). Functional diversity of farmland bees across rural–urban landscapes in a tropical megacity. *Ecological Applications*, *32*(8), e2699. <https://doi.org/10.1002/eap.2699>

Martins, K. T., Gonzalez, A., & Lechowicz, M. J. (2015). Pollination services are mediated by bee functional diversity and landscape context. *Agriculture, Ecosystems & Environment*, *200*, 12–20. <https://doi.org/10.1016/j.agee.2014.10.018>

Mazzeo, N. M., & Torretta, J. P. (2015). Wild bees (Hymenoptera: Apoidea) in an urban botanical garden in Buenos Aires, Argentina. *Studies on Neotropical Fauna and Environment*, *50*(3), 182–193. <https://doi.org/10.1080/01650521.2015.1093764>

McCravy, K., Geroff, R., & Gibbs, J. (2019a). Bee (Hymenoptera: Apoidea: Anthophila) Functional Traits in Relation to Sampling Methodology in a Restored Tallgrass Prairie. *Florida Entomologist*, *102*(1), 134. <https://doi.org/10.1653/024.102.0122>

McCravy, K., Geroff, R., & Gibbs, J. (2019b). Bee (Hymenoptera: Apoidea: Anthophila) Functional Traits in Relation to Sampling Methodology in a Restored Tallgrass Prairie. *Florida Entomologist*, *102*(1), 134. <https://doi.org/10.1653/024.102.0122>

Montoya‐Pfeiffer, P. M., Rodrigues, R. R., & Alves dos Santos, I. (2020). Bee pollinator functional responses and functional effects in restored tropical forests. *Ecological Applications*, *30*(3), e02054. <https://doi.org/10.1002/eap.2054>

Morales-Alba, A., Carvajal-Cogollo, J., & Morales, I. (2021). Abejas en sistemas agrícolas: Revisión de la diversidad taxonómica y funcional, y perspectivas de investigación. *Acta Biológica Colombiana*, *27*(2), 282–291. <https://doi.org/10.15446/abc.v27n2.92192>

Moretti, M., de Bello, F., Roberts, S. P. M., & Potts, S. G. (2009). Taxonomical vs. Functional responses of bee communities to fire in two contrasting climatic regions. *Journal of Animal Ecology*, *78*(1), 98–108. <https://doi.org/10.1111/j.1365-2656.2008.01462.x>

Munyuli, T. (2014). Influence of functional traits on foraging behaviour and pollination efficiency of wild social and solitary bees visiting coffee ( *Coffea canephora* ) flowers in Uganda. *Grana*, *53*(1), 69–89. <https://doi.org/10.1080/00173134.2013.853831>

Nooten, S. S., Odanaka, K., & Rehan, S. M. (2020). Characterization of wild bee communities in apple and blueberry orchards. *Agricultural and Forest Entomology*, *22*(2), 157–168. <https://doi.org/10.1111/afe.12370>

Normandin, É., Vereecken, N. J., Buddle, C. M., & Fournier, V. (2017). Taxonomic and functional trait diversity of wild bees in different urban settings. *PeerJ*, *5*, e3051. <https://doi.org/10.7717/peerj.3051>

O’Brien, C., & Arathi, H. S. (2019). Bee diversity and abundance on flowers of industrial hemp (Cannabis sativa L.). *Biomass and Bioenergy*, *122*, 331–335. <https://doi.org/10.1016/j.biombioe.2019.01.015>

Ockermüller, E., Kratschmer, S., Hainz-Renetzeder, C., Sauberer, N., Meimberg, H., Frank, T., Pascher, K., & Pachinger, B. (2023). Agricultural land-use and landscape composition: Response of wild bee species in relation to their characteristic traits. *Agriculture, Ecosystems & Environment*, *353*, 108540. <https://doi.org/10.1016/j.agee.2023.108540>

Odanaka, K. A., & Rehan, S. M. (2019). Impact indicators: Effects of land use management on functional trait and phylogenetic diversity of wild bees. *Agriculture, Ecosystems & Environment*, *286*, 106663. <https://doi.org/10.1016/j.agee.2019.106663>

Papanikolaou, A. D., Kühn, I., Frenzel, M., Kuhlmann, M., Poschlod, P., Potts, S. G., Roberts, S. P. M., & Schweiger, O. (2017). Wild bee and floral diversity co-vary in response to the direct and indirect impacts of land use. *Ecosphere*, *8*(11), e02008. <https://doi.org/10.1002/ecs2.2008>

Passaseo, A., Pétremand, G., Rochefort, S., & Castella, E. (2020). Pollinator emerging from extensive green roofs: Wild bees (Hymenoptera, Antophila) and hoverflies (Diptera, Syrphidae) in Geneva (Switzerland). *Urban Ecosystems*, *23*(5), 1079–1086. <https://doi.org/10.1007/s11252-020-00973-9>

Pei, C. K., Hovick, T. J., Duquette, C. A., Limb, R. F., Harmon, J. P., & Geaumont, B. A. (2022). Two common bee-sampling methods reflect different assemblages of the bee (Hymenoptera: Apoidea) community in mixed-grass prairie systems and are dependent on surrounding floral resource availability. *Journal of Insect Conservation*, *26*(1), 69–83. <https://doi.org/10.1007/s10841-021-00362-3>

Pei, C. K., Hovick, T. J., Limb, R. F., Harmon, J. P., & Geaumont, B. A. (2023). Invasive grass and litter accumulation constrain bee and plant diversity in altered grasslands. *Global Ecology and Conservation*, *41*, e02352. <https://doi.org/10.1016/j.gecco.2022.e02352>

Persson, A. S., Rundlöf, M., Clough, Y., & Smith, H. G. (2015). Bumble bees show trait-dependent vulnerability to landscape simplification. *Biodiversity and Conservation*, *24*(14), 3469–3489. <https://doi.org/10.1007/s10531-015-1008-3>

Peters, M. K., Peisker, J., Steffan-Dewenter, I., & Hoiss, B. (2016). Morphological traits are linked to the cold performance and distribution of bees along elevational gradients. *Journal of Biogeography*, *43*(10), 2040–2049. <https://doi.org/10.1111/jbi.12768>

Phillips, B. B., Williams, A., Osborne, J. L., & Shaw, R. F. (2018). Shared traits make flies and bees effective pollinators of oilseed rape (Brassica napus L.). *Basic and Applied Ecology*, *32*, 66–76. <https://doi.org/10.1016/j.baae.2018.06.004>

Pisanty, G., & Mandelik, Y. (2015). Profiling crop pollinators: Life history traits predict habitat use and crop visitation by Mediterranean wild bees. *Ecological Applications*, *25*(3), 742–752. <https://doi.org/10.1890/14-0910.1>

Ponisio, L. C., de Valpine, P., M’Gonigle, L. K., & Kremen, C. (2019). Proximity of restored hedgerows interacts with local floral diversity and species’ traits to shape long‐term pollinator metacommunity dynamics. *Ecology Letters*, *22*(7), 1048–1060. <https://doi.org/10.1111/ele.13257>

Poulsen, N. R., & Rasmussen, C. (2020). Island bees: Do wood nesting bees have better island dispersal abilities? *Apidologie*, *51*(6), 1006–1017. <https://doi.org/10.1007/s13592-020-00778-x>

Querejeta, M., Marchal, L., Pfeiffer, P., Roncoroni, M., Bretagnolle, V., Gaba, S., & Boyer, S. (2023). Environmental variables and species traits as drivers of wild bee pollination in intensive agroecosystems—A metabarcoding approach. *Environmental DNA*, *5*(5), 1078–1091. <https://doi.org/10.1002/edn3.421>

Rader, R., Bartomeus, I., Tylianakis, J. M., & Laliberté, E. (2014). The winners and losers of land use intensification: Pollinator community disassembly is non-random and alters functional diversity. *Diversity and Distributions*, *20*(8), 908–917. <https://doi.org/10.1111/ddi.12221>

Raiol, R. L., Gastauer, M., Campbell, A. J., Borges, R. C., Awade, M., & Giannini, T. C. (2021). Specialist Bee Species Are Larger and Less Phylogenetically Distinct Than Generalists in Tropical Plant–Bee Interaction Networks. *Frontiers in Ecology and Evolution*, *9*, 699649. <https://doi.org/10.3389/fevo.2021.699649>

Ramírez, S. R., Hernández, C., Link, A., & López-Uribe, M. M. (2015a). Seasonal cycles, phylogenetic assembly, and functional diversity of orchid bee communities. *Ecology and Evolution*, *5*(9), 1896–1907. <https://doi.org/10.1002/ece3.1466>

Ramírez, S. R., Hernández, C., Link, A., & López-Uribe, M. M. (2015b). Seasonal cycles, phylogenetic assembly, and functional diversity of orchid bee communities. *Ecology and Evolution*, *5*(9), 1896–1907. <https://doi.org/10.1002/ece3.1466>

Ramos-Fabiel, M. A., Pérez-García, E. A., González, E. J., Yáñez-Ordoñez, O., & Meave, J. A. (2019). Successional dynamics of the bee community in a tropical dry forest: Insights from taxonomy and functional ecology. *Biotropica*, *51*(1), 62–74. <https://doi.org/10.1111/btp.12619>

Rhoades, P., Griswold, T., Waits, L., Bosque-Pérez, N. A., Kennedy, C. M., & Eigenbrode, S. D. (2017). Sampling technique affects detection of habitat factors influencing wild bee communities. *Journal of Insect Conservation*, *21*(4), 703–714. <https://doi.org/10.1007/s10841-017-0013-0>

Ribeiro, C., Varassin, I. G., Pagioro, T. A., & Souza, J. M. T. D. (2024). Bee and plant traits drive temporal similarity of pollination interactions in areas under distinct restoration strategies. *Arthropod-Plant Interactions*. <https://doi.org/10.1007/s11829-024-10064-7>

Ricotta, C., & Moretti, M. (2011). CWM and Rao’s quadratic diversity: A unified framework for functional ecology. *Oecologia*, *167*(1), 181–188. <https://doi.org/10.1007/s00442-011-1965-5>

Rodríguez S, S., Pérez-Giraldo, L. C., Vergara, P. M., Carvajal, M. A., & Alaniz, A. J. (2021). Native bees in Mediterranean semi-arid agroecosystems: Unravelling the effects of biophysical habitat, floral resource, and honeybees. *Agriculture, Ecosystems & Environment*, *307*, 107188. <https://doi.org/10.1016/j.agee.2020.107188>

Rollin, O., Bretagnolle, V., Fortel, L., Guilbaud, L., & Henry, M. (2015). Habitat, spatial and temporal drivers of diversity patterns in a wild bee assemblage. *Biodiversity and Conservation*, *24*(5), 1195–1214. <https://doi.org/10.1007/s10531-014-0852-x>

Roquer‐Beni, L., Alins, G., Arnan, X., Boreux, V., García, D., Hambäck, P. A., Happe, A., Klein, A., Miñarro, M., Mody, K., Porcel, M., Rodrigo, A., Samnegård, U., Tasin, M., & Bosch, J. (2021). Management‐dependent effects of pollinator functional diversity on apple pollination services: A response–effect trait approach. *Journal of Applied Ecology*, *58*(12), 2843–2853. <https://doi.org/10.1111/1365-2664.14022>

Samnegård, U., Hambäck, P. A., Eardley, C., Nemomissa, S., & Hylander, K. (2015). Turnover in bee species composition and functional trait distributions between seasons in a tropical agricultural landscape. *Agriculture, Ecosystems & Environment*, *211*, 185–194. <https://doi.org/10.1016/j.agee.2015.06.010>

Schmolke, A., Galic, N., & Hinarejos, S. (2023). SolBeePop: A model of solitary bee populations in agricultural landscapes. *Journal of Applied Ecology*, *60*(12), 2573–2585. <https://doi.org/10.1111/1365-2664.14541>

Shi, X., Axmacher, J. C., Chong, H., Xiao, H., Luo, S., Xu, H., Li, W., & Zou, Y. (2022). Effects of farmland consolidation in southern China on wild bee species composition, nesting location and body size variations. *Agricultural and Forest Entomology*, *24*(3), 371–379. <https://doi.org/10.1111/afe.12500>

Smith, C., Weinman, L., Gibbs, J., & Winfree, R. (2019). Specialist foragers in forest bee communities are small, social or emerge early. *Journal of Animal Ecology*, *88*(8), 1158–1167. <https://doi.org/10.1111/1365-2656.13003>

Sobieraj‐Betlińska, A., Szefer, P., & Twerd, L. (2022). Linear woodlots increase wild bee abundance by providing additional food sources in an agricultural landscape. *Agricultural and Forest Entomology*, afe.12529. <https://doi.org/10.1111/afe.12529>

Staton, T., Walters, R. J., Breeze, T. D., Smith, J., & Girling, R. D. (2022). Niche complementarity drives increases in pollinator functional diversity in diversified agroforestry systems. *Agriculture, Ecosystems & Environment*, *336*, 108035. <https://doi.org/10.1016/j.agee.2022.108035>

Steffan-Dewenter, I., & Tscharntke, T. (2001). Succession of bee communities on fallows. *Ecography*, *24*(1), 83–93. <https://doi.org/10.1034/j.1600-0587.2001.240110.x>

Steinert, M., Sydenham, M. A. K., Eldegard, K., & Moe, S. R. (2020). Conservation of solitary bees in power-line clearings: Sustained increase in habitat quality through woody debris removal. *Global Ecology and Conservation*, *21*, e00823. <https://doi.org/10.1016/j.gecco.2019.e00823>

Stemkovski, M., Pearse, W. D., Griffin, S. R., Pardee, G. L., Gibbs, J., Griswold, T., Neff, J. L., Oram, R., Rightmyer, M. G., Sheffield, C. S., Wright, K., Inouye, B. D., Inouye, D. W., & Irwin, R. E. (2020). Bee phenology is predicted by climatic variation and functional traits. *Ecology Letters*, *23*(11), 1589–1598. <https://doi.org/10.1111/ele.13583>

Sydenham, M. A. K., Moe, S. R., Stanescu‐Yadav, D. N., Totland, Ø., & Eldegard, K. (2016). The effects of habitat management on the species, phylogenetic and functional diversity of bees are modified by the environmental context. *Ecology and Evolution*, *6*(4), 961–973. <https://doi.org/10.1002/ece3.1963>

Sydenham, M. A. K., Moe, S. R., Totland, Ø., & Eldegard, K. (2015). Does multi‐level environmental filtering determine the functional and phylogenetic composition of wild bee species assemblages? *Ecography*, *38*, 140–153. <https://doi.org/10.1111/ecog.00938>

Tonietto, R. K., Ascher, J. S., & Larkin, D. J. (2017). Bee communities along a prairie restoration chronosequence: Similar abundance and diversity, distinct composition. *Ecological Applications*, *27*(3), 705–717. <https://doi.org/10.1002/eap.1481>

Török, E., Gallé, R., & Batáry, P. (2022). Fragmentation of forest-steppe predicts functional community composition of wild bee and wasp communities. *Global Ecology and Conservation*, *33*, e01988. <https://doi.org/10.1016/j.gecco.2021.e01988>

Turo, K. J., Spring, M. R., Sivakoff, F. S., Delgado de la flor, Y. A., & Gardiner, M. M. (2021). Conservation in post‐industrial cities: How does vacant land management and landscape configuration influence urban bees? *Journal of Applied Ecology*, *58*(1), 58–69. <https://doi.org/10.1111/1365-2664.13773>

Villalobos, S., & Vamosi, J. C. (2018). Climate and habitat influences on bee community structure in Western Canada. *Canadian Journal of Zoology*, *96*(9), 1002–1009. <https://doi.org/10.1139/cjz-2017-0226>

Villalta, I., Bouget, C., Lopez-Vaamonde, C., & Baude, M. (2022). Phylogenetic, functional and taxonomic responses of wild bee communities along urbanisation gradients. *Science of The Total Environment*, *832*, 154926. <https://doi.org/10.1016/j.scitotenv.2022.154926>

Weber, M., Diekötter, T., Dietzsch, A. C., Erler, S., Greil, H., Jütte, T., Krahner, A., & Pistorius, J. (2023). Urban wild bees benefit from flower-rich anthropogenic land use depending on bee trait and scale. *Landscape Ecology*, *38*(11), 2981–2999. <https://doi.org/10.1007/s10980-023-01755-2>

Williams, N. M., Crone, E. E., Roulston, T. H., Minckley, R. L., Packer, L., & Potts, S. G. (2010). Ecological and life-history traits predict bee species responses to environmental disturbances. *Biological Conservation*, *143*(10), 2280–2291. <https://doi.org/10.1016/j.biocon.2010.03.024>

Wilson, C. J., & Jamieson, M. A. (2019). The effects of urbanization on bee communities depends on floral resource availability and bee functional traits. *PLOS ONE*, *14*(12), e0225852. <https://doi.org/10.1371/journal.pone.0225852>

Woodcock, B. A., Garratt, M. P. D., Powney, G. D., Shaw, R. F., Osborne, J. L., Soroka, J., Lindström, S. A. M., Stanley, D., Ouvrard, P., Edwards, M. E., Jauker, F., McCracken, M. E., Zou, Y., Potts, S. G., Rundlöf, M., Noriega, J. A., Greenop, A., Smith, H. G., Bommarco, R., … Pywell, R. F. (2019). Meta-analysis reveals that pollinator functional diversity and abundance enhance crop pollination and yield. *Nature Communications*, *10*(1), 1481. <https://doi.org/10.1038/s41467-019-09393-6>

Woodcock, B. A., Harrower, C., Redhead, J., Edwards, M., Vanbergen, A. J., Heard, M. S., Roy, D. B., & Pywell, R. F. (2014). National patterns of functional diversity and redundancy in predatory ground beetles and bees associated with key UK arable crops. *Journal of Applied Ecology*, *51*(1), 142–151. <https://doi.org/10.1111/1365-2664.12171>

Wray, J. C., Neame, L. A., & Elle, E. (2014a). Floral resources, body size, and surrounding landscape influence bee community assemblages in oak-savannah fragments. *Ecological Entomology*, *39*(1), 83–93. <https://doi.org/10.1111/een.12070>

Wray, J. C., Neame, L. A., & Elle, E. (2014b). Floral resources, body size, and surrounding landscape influence bee community assemblages in oak-savannah fragments: Bee communities in oak-savannah fragments. *Ecological Entomology*, *39*(1), 83–93. <https://doi.org/10.1111/een.12070>

Wu, P., Axmacher, J. C., Song, X., Zhang, X., Xu, H., Chen, C., Yu, Z., & Liu, Y. (2018). Effects of Plant Diversity, Vegetation Composition, and Habitat Type on Different Functional Trait Groups of Wild Bees in Rural Beijing. *Journal of Insect Science*, *18*(4). <https://doi.org/10.1093/jisesa/iey065>

Wyver, C., Potts, S. G., Edwards, M., Edwards, R., Roberts, S., & Senapathi, D. (2023). Climate-driven phenological shifts in emergence dates of British bees. *Ecology and Evolution*, *13*(7), e10284. <https://doi.org/10.1002/ece3.10284>

Xie, T., Orr, M. C., Zhang, D., Ferrari, R. R., Li, Y., Liu, X., Niu, Z., Wang, M., Zhou, Q., Hao, J., Zhu, C., & Chesters, D. (2023). Phylogeny-based assignment of functional traits to DNA barcodes outperforms distance-based, in a comparison of approaches. *Molecular Ecology Resources*, *23*(7), 1526–1539. <https://doi.org/10.1111/1755-0998.13813>
